# PROLONGED EFFECTS OF THE COVID-19 PANDEMIC ON FRAILTY TRENDS IN A VETERAN SUBPOPULATION

**DOI:** 10.1093/geroni/igae098.4058

**Published:** 2024-12-31

**Authors:** Nalini Bhalla, Janet Fawcett

**Affiliations:** Phoenix VA Medical Center, Phoenix, Arizona, United States; Phoenix VA Health Care System, Phoenix, Arizona, United States

## Abstract

The first year of the Covid-19 pandemic resulted in an increase in frailty when compared to a recovering trend the prior year in our previously studied subpopulation of 3532 older veterans. We used an automatically generated Veterans Affairs (VA) Metrics computed assessment score to monitor frailty trends for an additional two years to see if this increase persists. We studied the one year difference in mortality Care Assessment Need (CAN) scores in 2652 veterans (mean(SD) age 71.7(1.3) years, 98.8% Male) from 2019 to 2023. Inclusion criteria were age 70-75 and CAN score >=75 as of 2/8/19. Veterans who died during the study period or did not have a 1-year mortality CAN score for any of the years were excluded. The year to year difference between the mortality CAN scores was compared with a one-way ANOVA with repeated measures with a Greenhouse-Geisser correction and found to be significant for increasing frailty (p< 0.001). We also compared the last mean (SD) CAN score from the 2652 patients alive on 2/10/23 with the 735 patients deceased between 2/12/21 and 2/10/23 and found a significantly higher CAN score in the patients who died (p< 0.001). The sequelae of repeated Covid infections involve a broad array of organ systems with an unclear duration of excess risk. Additionally, an uneven post pandemic recovery in the diagnosis and management of chronic conditions may explain the persistent increase in frailty in our studied veterans up to 3 years after the pandemic was declared.

